# PSE: A tool for browsing a large amount of MEDLINE/PubMed abstracts with gene names and common words as the keywords

**DOI:** 10.1186/1471-2105-6-295

**Published:** 2005-12-10

**Authors:** Takashi Yoneya

**Affiliations:** 1 Pharmaceutical Research Laboratories, Pharmaceutical Division, Kirin Brewery Co. Ltd., 3 Miyahara, Takasaki, Gunma 370-1295, Japan

## Abstract

**Background:**

MEDLINE/PubMed (hereinafter called PubMed) is one of the most important literature databases for the biological and medical sciences, but it is impossible to read all related records due to the sheer size of the repository. We usually have to repeatedly enter keywords in a trial-and-error manner to extract useful records. Software which can reduce such a laborious task is therefore required.

**Results:**

We developed a web-based software, the PubMed Sentence Extractor (PSE), which parses large number of PubMed abstracts, extracts and displays the co-occurrence sentences of gene names and other keywords, and some information from EntrezGene records. The result links to whole abstracts and other resources such as the Online Mendelian Inheritance in Men and Reference Sequence. While PSE executes at the sentence-level when evaluating the existence of keywords, the popular PubMed operates at the record-level. Therefore, the relationship between the two keywords, a gene name and a common word, is more accurately captured by PSE than PubMed. In addition, PSE shows the list of keywords and considers the synonyms and variations on gene names. Through these functions, PSE would reduce the task of searching through records for gene information.

**Conclusion:**

We developed PSE in order to extract useful records efficiently from PubMed. This system has four advantages over a simple PubMed search; the reduction in the amount of collected literatures, the showing of keyword lists, the consideration for synonyms and variations on gene names, and the links to external databases. We believe PSE is helpful in collecting necessary literatures efficiently in order to find research targets. PSE is freely available under the GPL licence as additional files to this manuscript.

## Background

Recent progress in sequence technology has revealed whole genome sequences of various species, such as human and mouse, leading to the production of comprehensive databases of gene sequences. Elucidation of gene functions is one of the most important issues in the so-called post-genomic sequence era and many scientists are actively tackling it. Although some results are presented as numeric or character string data, most of the results are published in literatures. Projects which aim to improve the usability of gene information include Gene Ontology (GO) [1] and Reference Sequence (RefSeq) [2]. Steady progress with principal parts of these projects are due to the manual curations by many experts. The goal of the GO project is to produce controlled vocabularies which can then be used to classify genes automatically. RefSeq aims to provide a comprehensive and non-redundant database containing not only sequences but also other related information. Although these secondary or higher-level databases are very useful to survey the information of many genes, the daily incorporation of all publicized data into the databases is impossible. We therefore need to use primary databases such as PubMed [3] to get the latest information. Many recent approaches using natural language processing techniques have been reported [4-6]. PubMed, which contains abstracts and other bibliographic information, is one of the most important literature databases for the biological and medical sciences. However, it is very large in size, containing over 12 million citations. Reading all related abstracts in such a huge database is almost impossible. We usually have to repeatedly enter keywords in a trial-and-error manner when looking for useful records. In order to collect necessary literatures efficiently, new software is required.

We developed new software to address this requirement based on the following strategies. First, most of the abstracts contain the important sentences which capture the main points of their respective article. Second, in many cases, the importance is decided by the relationship between gene names and common words. For example:

c-Kit is constitutively activated in various tumors.

c-Kit expression increases in various tumors.

c-Kit expression is observed in various tumors.

Although all of these sentences indicate the relationship between c-Kit (a kind of receptor-type protein tyrosine kinase) and various tumors, the relative importance of their meanings is different and determined by the underlined words. We presume that extracting and reading the important sentences prior to reading whole abstracts would reduce the task of literature searching. One noteworthy point is that the importance of each sentence depends on the reader's interest. If the reader is interested in the development of an anti-cancer drug which functions as the inhibitor of c-Kit, the first sentence would be more important than the second one because the drug should not achieve an anti-tumor effect if c-Kit is not active. On the other hand, if the reader wants to develop antibody-based drugs which target c-Kit, the second sentence would be more important than the first because the number of molecules on the cell surface should be more crucial than whether it is active or not. Although this example shows the difference of predicator, some noun (e.g. tissue name or disease name) might be important for another user. From another viewpoint, a sentence that reports experimental result might be important for one user, but another one that describes the background might be of interest to another user. Thus, the importance of each sentence depends on the user's interest. In this report, we introduce a web-based software, the PubMed Sentence Extractor (PSE), which parses huge PubMed abstracts, and then extracts and displays the co-occurrence sentences of gene names with other keywords.

## Implementation

### Keyword extraction

PSE identifies words as keywords in sentences with gene names after filtering out the less valuable words. The filtering is carried out by using a stopword list based on statistical and heuristic methods. The list is complied using a metric commonly found in information retrieval systems combined with a method described below.

24,126 PubMed abstracts were retrieved using "result" as a keyword because it was postulated not to be biased in particular fields of articles. The term frequency (tf) for word i in abstract j (tf(i,j)) is calculated as follows:

tf(i,j)=frequency of word i in abstract jnumber of all words in abstract j
 MathType@MTEF@5@5@+=feaafiart1ev1aaatCvAUfKttLearuWrP9MDH5MBPbIqV92AaeXatLxBI9gBaebbnrfifHhDYfgasaacH8akY=wiFfYdH8Gipec8Eeeu0xXdbba9frFj0=OqFfea0dXdd9vqai=hGuQ8kuc9pgc9s8qqaq=dirpe0xb9q8qiLsFr0=vr0=vr0dc8meaabaqaciGacaGaaeqabaqabeGadaaakeaacqWG0baDcqWGMbGzdaqadaqaaiabdMgaPjabcYcaSiabdQgaQbGaayjkaiaawMcaaiabg2da9maalaaabaGaeeOzayMaeeOCaiNaeeyzauMaeeyCaeNaeeyDauNaeeyzauMaeeOBa4Maee4yamMaeeyEaKNaeeiiaaIaee4Ba8MaeeOzayMaeeiiaaIaee4DaCNaee4Ba8MaeeOCaiNaeeizaqMaeeiiaaIaeeyAaKMaeeiiaaIaeeyAaKMaeeOBa4MaeeiiaaIaeeyyaeMaeeOyaiMaee4CamNaeeiDaqNaeeOCaiNaeeyyaeMaee4yamMaeeiDaqNaeeiiaaIaeeOAaOgabaGaeeOBa4MaeeyDauNaeeyBa0MaeeOyaiMaeeyzauMaeeOCaiNaeeiiaaIaee4Ba8MaeeOzayMaeeiiaaIaeeyyaeMaeeiBaWMaeeiBaWMaeeiiaaIaee4DaCNaee4Ba8MaeeOCaiNaeeizaqMaee4CamNaeeiiaaIaeeyAaKMaeeOBa4MaeeiiaaIaeeyyaeMaeeOyaiMaee4CamNaeeiDaqNaeeOCaiNaeeyyaeMaee4yamMaeeiDaqNaeeiiaaIaeeOAaOgaaaaa@88F1@

Inverse document frequency (idf) for word i (idf(i)) is calculated as follows:

idf(i)=log⁡enumber of total abstractsnumber of abstracts containing word i
 MathType@MTEF@5@5@+=feaafiart1ev1aaatCvAUfKttLearuWrP9MDH5MBPbIqV92AaeXatLxBI9gBaebbnrfifHhDYfgasaacH8akY=wiFfYdH8Gipec8Eeeu0xXdbba9frFj0=OqFfea0dXdd9vqai=hGuQ8kuc9pgc9s8qqaq=dirpe0xb9q8qiLsFr0=vr0=vr0dc8meaabaqaciGacaGaaeqabaqabeGadaaakeaacqWGPbqAcqWGKbazcqWGMbGzdaqadaqaaiabdMgaPbGaayjkaiaawMcaaiabg2da9iGbcYgaSjabc+gaVjabcEgaNnaaBaaaleaacqWGLbqzaeqaaOWaaSaaaeGabaqp9labb6gaUjabbwha1jabb2gaTjabbkgaIjabbwgaLjabbkhaYjabbccaGiabb+gaVjabbAgaMjabbccaGiabbsha0jabb+gaVjabbsha0jabbggaHjabbYgaSjabbccaGiabbggaHjabbkgaIjabbohaZjabbsha0jabbkhaYjabbggaHjabbogaJjabbsha0jabbohaZbqaaiabb6gaUjabbwha1jabb2gaTjabbkgaIjabbwgaLjabbkhaYjabbccaGiabb+gaVjabbAgaMjabbccaGiabbggaHjabbkgaIjabbohaZjabbsha0jabbkhaYjabbggaHjabbogaJjabbsha0jabbohaZjabbccaGiabbogaJjabb+gaVjabb6gaUjabbsha0jabbggaHjabbMgaPjabb6gaUjabbMgaPjabb6gaUjabbEgaNjabbccaGiabbEha3jabb+gaVjabbkhaYjabbsgaKjabbccaGiabbMgaPbaaaaa@8BB9@

The term frequency – inverse document frequency (tf-idf) score for word i (tf-idf(i)) is calculated using the maximum value of tf(i,j) according to the following equation:

*tf *- *idf*(*i*) = *max_j _*(tf(i,j)) × idf(i)

The words found in more than 0.1% of the abstracts and whose tf-idf scores were less than 0.1 were saved. Next, the words included in more than 1% of the abstracts and whose tf-idf scores were more than 0.1 were listed. The words suited for keywords in the latter list were removed manually and the remaining list was saved. The two lists were merged and saved as the stopword list (SL1). SL1 contains 8,814 words. SL1 is used for both the creation of a gene name dictionary and the extraction of common words.

After establishing SL1, each word in the abstracts is compared with SL1. If the word is not included in SL1, the occurrence is counted, recorded and displayed as a pull-down list. The counts of some plural forms obtained by adding a final "s" are merged with their singular forms. Twenty to twenty-five percent of the total words are selected as keywords with SL1 if PSE searches whole sentences (data not shown). To reduce the number of keywords, PSE only searches sentences which contain gene names as keywords.

### Compilation of a gene name dictionary

PSE first extracts sentences containing gene names by using a dictionary which was created without any learning algorithm to properly evaluate whether each word or phrase is a gene name or not. This key component of our system was compiled from EntrezGene (formerly LocusLink) records [2]. Two stopword lists (SL1 and SL2) were used for reducing the number of false positives and expanding aliases of gene names to improve recall in the compiling process, as detailed below.

Gene name information was extracted from EntrezGene records. The records which have "Official Symbol" and originating from human, mouse and rat were used. Aliases and symbols were extracted from each record and a series of aliases were created based on several rules. For example: (a) add/remove hyphen/space, convert Arabic number into Roman number if the name ends with a number (e.g. Akt-1 => Akt1, Akt-1, AktI, Akt-I, Akt I), (b) trim the name if it contains some specific words (e.g. if one of the words is "isoform", then "fibulin 1 isoform A" => "fibulin 1"). Then, each word or phrase was compared with SL1 and another stopword list (SL2) described below, and the word or phrase, which did not match with one or both of them, was added as an alias after being converted into uppercase. One final consideration is the homonymous gene names of common words, e.g. yes, which generate many false positives. Therefore, elimination of homonyms for common words is important. Three sets of abstracts containing around 1,500 records were retrieved using "cancer AND kinase", "autoimmune" and "hypertension" as keywords, respectively, and the candidate words of gene names were marked. Browsing the results, we created two additional lists, an ordinary stopword list (SL2) and an adjacent pattern list (PL1), based on the incorrect assessments. SL2 contains 304 words and phrases, and PL1 contains 231 words and suffixes. SL1 and SL2 are used for compiling the dictionary and PL1 is used for the actual extraction from the PubMed abstracts.

### Gene name extraction

The three steps (tokenization, elimination of stopwords in case of single words and comparison with the gene name dictionary) are carried out during gene name extraction. Prior to these steps, additional stopwords are extracted from each abstract with the aim of reducing the number of false positives caused by the acronyms of some proper names, e g. chemical compounds and diseases. These stopwords are only applied to each abstract to avoid reducing the recall. The detail is as follows.

Each abstract is split into sentences and stopwords are extracted first. If a word is in parentheses or located between two commas, the adjacent word is compared with PL1. This comparison is carried out using a backward match. For example, if "OSUS" is in PL1 and the phrase, "systematic lupus erythematosus (SLE) ", is found in the current sentence. Because the terminus of "erythematosus" matches with "OSUS", the word, "SLE", is ruled out as a candidate for a gene name. This process is effective to remove many acronyms of chemical compounds, disease names and so on. The exclusion using PL1 is applied to each abstract independently. In contrast, SL1 and SL2 are applied to all records.

After the extraction of stopwords, gene name extraction is carried out. Starting with the top of the sentence, 6 continuous words are compared with the dictionary and are phased out if the phrase does not match. All letters are converted into uppercase prior to the comparison in case of more than two words. To improve the precision, the following rules are applied to single words; (a) a word containing non-alphabetical letters or more than one uppercase letters at the beginning of the sentence, or (b) a word that is not consisting entirely of lowercase letters and not located at the front of the sentence. If a word matches one of these rules, it is converted into uppercase and compared with the dictionary. Therefore, PSE cannot detect single words containing only lowercase letters as gene names.

### System implementation

PSE is written with Perl and PHP, and runs on UNIX. A user retrieves PubMed abstracts in XML format and registers them into the system. During the registration process, PSE splits the whole abstract into sentences, assigns each sentence an ID, counts the number of keywords, searches for gene names and records the sentence ID for each gene symbol. Although these steps appear to take time, the following process is so fast that users would not feel stress. Users select a gene symbol and a common word from pull-down menus, and PSE displays the extracted sentences and related information. Because these sentences and information are linked to whole abstracts and external web sites, such as EntrezGene, Refseq and OMIM [7], users can obtain more information on interesting genes and sentences. Although PSE has no rule for assigning homonyms, it can display a list of them in case of incorrect assignments. Using the list, users can select the correct symbol and get information on the correct gene. This program also contains a simple text search function. Therefore, users can search co-occurrence sentences with any keyword in the registered abstracts.

## Results and Discussion

### Detection sensitivity of gene names

The detection sensitivity of gene names of PSE was evaluated using two sets of forty abstracts randomly selected from the whole PubMed. The precision, recall and F-measure were 77–78%, 45–66% and 57–71%, respectively (Ev1, Table 1). In order to collect an extensive and rich collection of mammalian gene names, we retrieved 4,548 abstracts using the following keywords, "gene AND disease AND activation". Two sets of forty abstracts were randomly selected from this set and used for another evaluation. The precision, recall and F-measure were 86–93%, 58–65% and 69–76%, respectively (Ev2, Table 1). PSE was also evaluated with the GENIA corpus [8]. The precision, recall and F-measure were 63%, 65% and 64%, respectively (Ev3, Table 1). Homonyms were not considered in this evaluation.

**Table 1:** Sensitivity of gene name extraction of PSE. Five datasets were used for the evaluation. The first two datasets represented as Set 1A, 1B contain forty abstracts retrieved with randomly generated PubMed IDs, respectively. The next two datasets labeled as Set 2A, 2B contain forty abstracts which were randomly selected from the 4,548 abstracts retrieved with "gene AND disease AND activation" as keywords, respectively. The last dataset represented as GENIA is the result from using the GENIA corpus containing 2,000 abstracts. The results using Set 1A,1B and Set 2A, 2B and GENIA are represented as Ev1, Ev2 and Ev3 in this manuscript, respectively. TP, FP and FN represent the true positive, the false positive and the false negative, respectively.

**Dataset**	**TP**	**FP**	**FN**	**Precision**	**Recall**	**F-measure**
Set 1A	50	15	61	76.9%	45.0%	56.8%
Set 1B	40	11	21	78.4%	65.6%	71.4%
Set 2A	287	23	157	92.6%	64.6%	76.1%
Set 2B	291	49	210	85.6%	58.1%	69.2%
GENIA	12,842	7,752	6,912	62.4%	65.0%	63.7%

### Exploring of cancer related genes

**Figure 1:**
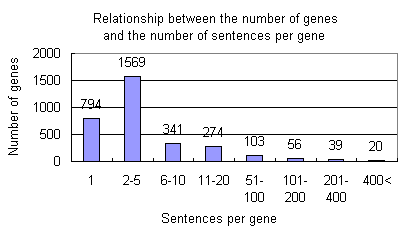
**Relationship between the number of genes and the number of the sentences per genes. **8,104 PubMed abstracts were retrieved using "cancer AND overexpression" as the keywords and registered into PSE. PSE detected 3,196 kinds of gene names. 794 gene names were included in only one sentence. Another 2,402 gene names were included in more than two sentences and 492 gene names were included in more than ten sentences.

**Figure 2:**
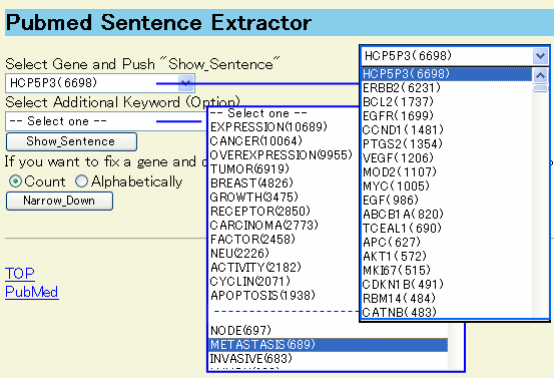
**The screen for selecting a gene name and a common word. **8,104 PubMed abstracts were retrieved using "cancer AND overexpression" as the keywords and registered into PSE in this example. The user selects a gene name and a common word in this screen.

To demonstrate the usefulness of this program, we show an example of exploring cancer related genes. 8,104 PubMed abstracts were retrieved using "cancer AND overexpression" as the keywords and registered into PSE. PSE found 3,196 gene names in the abstracts, of which 492 gene names were included in more than 10 sentences (Figure 1). Figure 2 is a screenshot of the page selecting a gene name and a common word. One pull-down list of genes displayed many well-known cancer-related genes with parentheses showing the number of sentences containing each gene name. Another one is the pull-down list of common words. It contained many words relating to the gene function, e.g. growth, apoptosis, metastasis and so on.

**Figure 3:**
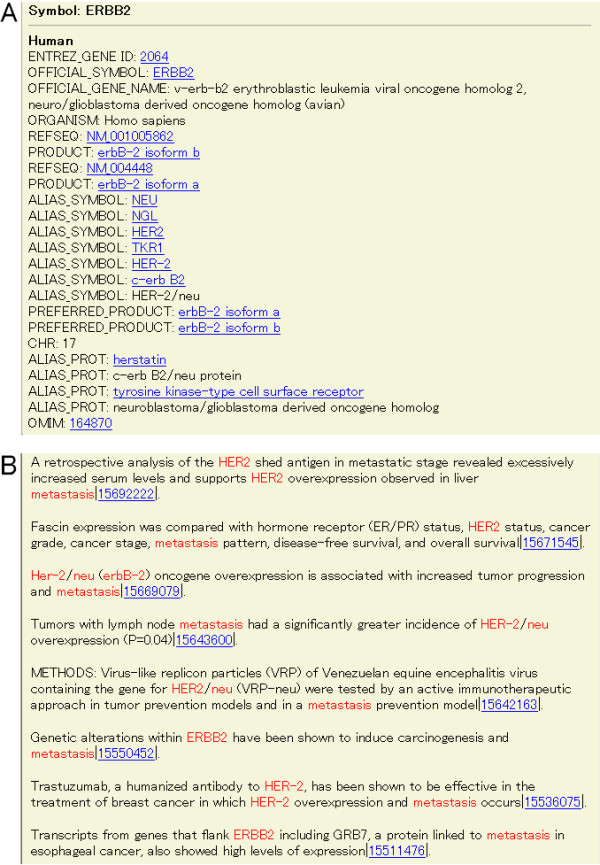
**The screen for selecting ERBB2 and metastasis as the keywords. **Figure 3 shows the result from selecting ERBB2 and metastasis in the screen shown as Figure 2. (A) The front part of the result shows information about the gene and the links to external resources, such as EntrezGene, OMIM and RefSeq. (B) The back part of the result shows the extracted sentences. The selected keywords including synonyms of ERBB2 are highlighted. The number at the end of each sentence is the PubMed ID and links to the whole abstract.

When ERBB2 and metastasis were selected in the screenshot shown in Figure 2, we automatically get the result shown in Figure 3. Figure 3A is the front part of the result. This part shows information about the gene and the links to the external resources, EntrezGene, OMIM and RefSeq. Figure 3B is the back part. The selected keywords including synonyms of ERBB2 are highlighted. The number at the end of each sentence is the PubMed ID that links to the whole abstract. Figure 3B indicates that occasionally, sentences are not relevant to the information being sought (e.g. the second sentence of this figure). Thus, while the user will still have to manually scan the result set, the aim of PSE is to shorten the sentences that the user will have to sift through. Users can obtain the information on the relationship of ERBB2 and metastasis even if they cannot come up with the words, ERBB2 and metastasis.

**Figure 4:**
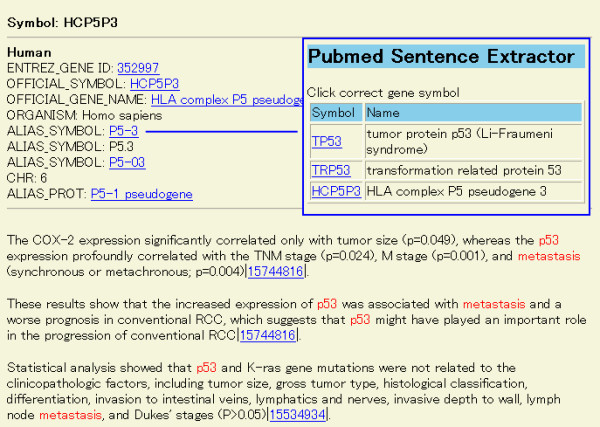
**The screen for selecting HCP5P3 and metastasis as keywords. **Figure 4 shows the result from selecting HCP5P3 and metastasis in the screen shown as Figure 2. Upper right is the window selecting a correct gene when the assignment is incorrect. TP53 is the correct gene in this example.

Figure 4 is the result when HCP5P3 and metastasis were selected from the page of Figure 2. Although the assignment of the gene symbol is incorrect, PSE displays other candidates by clicking one of the aliases. In this case, HCP5P3 is incorrectly assigned as a homonym and the correct symbol is TP53, one of the popular tumor suppressor genes. As a result, users can determine the relationship between TP53 and metastasis.

## Discussion

We developed a web-based text mining software for use within an intranet. Because the response time was few seconds under the following test condition, less than 10,000 abstracts using a standard PC, AMD AthlonXP 2500 and 512 Mb RAM, we believe that our software can be developed for actual use.

PSE has the following four advantages over a basic PubMed search;

1) PSE can extract sentences which imply gene function or its role, effectively, because PSE uses one gene name and one common word as two keywords. In addition, PSE evaluates the existence of keywords for individual sentences, but the popular PubMed search evaluates that at the record-level. Therefore, the relationship between the two keywords is considered to be more significant from the result of PSE than from PubMed. Because of these advantages, users can read the extracted sentences, and extrapolate relationships between genes and their functions or roles, prior to reading whole abstracts. It will reduce the overall searching task and help to get necessary records more efficiently.

2) PSE displays the lists of gene names and common words. There is a limit to the number of appropriate and various keywords thought by users, and this process fully depends on their knowledge and search technique. The lists are considered to be useful for them not only to select keywords but also to cover the knowledge and search skill. In addition, they may help users to find unexpected roles or functions of genes, and get new idea and strategies.

3) PSE considers synonyms and variations of gene names. Eight sentences were shown in Figure 3B. If only "ERBB2" was used as a search keyword, only two sentences would be extracted and the others would be missed. This example shows that consideration of synonyms and variation is a powerful function.

4) PSE links to external databases such as OMIM, EntrezGene and RefSeq. Because there are various well-organized and high-quality information in these databases, users can get an overview of each gene including sequence information. It is noteworthy that the link to sequence information would ease the combination with other systems, e.g. microarray, similarity search and so on.

Based on these four advantages, we believe that PSE is a useful tool for collecting literature information about gene efficiently.

In the previous version of PSE [9], we only used the dictionary to extract gene names. The new version of PSE employs the dictionary and additional rules, SL1, SL2 and PL1. Because the precision was significantly improved from 48% to 77% by this modification, the addition of other rules was quite effective. The choice of the dataset affects on the evaluation. The precision of Ev2 is better than Ev1 and the variation was caused by the difference in gene name frequency. It seems that Ev1 is fair but far from practical, but Ev2 is one of the most considerable examples to use. We think user would feel the latter sensitivity in the practical use. The precision of Ev3 is slightly worse than Ev1 and Ev2. This difference is caused by the discrepancy in criteria between GENIA and PSE, and the false negatives of GENIA. Assignment of homonyms is another important issue and is not yet regarded by our system.

## Conclusion

We developed PSE in order to extract useful records efficiently from PubMed while focusing on gene information. This system has the following four advantages: the reduction of the amount of records in the result set, the showing of keyword lists, the consideration for synonyms and variations in gene names, and the links to external databases. We believe that PSE is helpful in collecting useful literatures efficiently in order to find research targets. PSE is intended to be used in a small intranet and it is freely available under GPL licence as additional files to this manuscript.

## Availability and requirements

PSE is freely available under the GPL license as additional files to this manuscript [see Additional file 1]. It runs on UNIX platform and requires PHP, Perl and Apache.

## Authors' contributions

TY designed and programmed the software and wrote this manuscript.

## Supplementary Material

Additional File 1 **PSE package**. This gzip compressed tar archive is a complete PSE package. It includes the programs, instruction and sample data. Click here for file
