# Supplementary material for: PSE: A tool for browsing a large amount of MEDLINE/PubMed abstracts with gene names and common words as the keywords
Source: BMC Bioinformatics. 2005 Dec 10;6:295. doi: 10.1186/1471-2105-6-295 (PMC1326231; doi:10.1186/1471-2105-6-295)
Supplement: Additional File 1 — PSE package. This gzip compressed tar archive is a complete PSE package. It includes the programs, instruction and sample data. [file 1471-2105-6-295-S1.gz › PSE/README.doc]

## LICENCE

PSE (Pubmed Sentence Extractor) version 1.1, Copyright (C) 20/March/2005 Takashi Yoneya

This program is free software; you can redistribute it and/or modify it under the terms of the GNU General Public License as published by the Free Software Foundation; either version 2 of the License, or (at your option) any later version.

This program is distributed in the hope that it will be useful, but WITHOUT ANY WARRANTY; without even the implied warranty of MERCHANTABILITY or FITNESS FOR A PARTICULAR PURPOSE. See the GNU General Public License for more details.

You should have received a copy of the GNU General Public License along with this program; if not, write to the Free Software Foundation, Inc., 59 Temple Place - Suite 330, Boston, MA 02111-1307, USA.

## INSTALL

- Decompress the archives "PSE.tar.gz".
- A folder, "PSE", containing 11 files, including this file, will be created.
- Download and decompress the EntrezGene file and place in the PSE folder.
  - (EntrezGene :ftp://ftp.ncbi.nlm.nih.gov/refseq/LocusLink/ARCHIVE/LL_tmpl.gz )
  - Attention!! Don't change the name, LL_tmpl.
- Change the permission of all the files and PSE directory to 777.
- Type "perl install_dictionary.pl" at PSE directory. This process takes for a while and following 9 files will be created.
  - LL_ID, LL_hmr, LL_human, LL_mouse, LL_rat, alias.dat, symbol.dat, text.count, homonym
- Setup the http server and check the top page (index.php) is correctly displayed.
  - Check php.ini and confirm the assignment of enough memory and process time to PHP
  - Confirm “register_globals = On” of php.ini

### TUTORIAL

**Data Registration**

- Download the Pubmed abstracts as XML format or use sample.xml in PSE directory.　The sample.xml is used in this tutorial.
-

- Confirm XML format, Total record < 10,000 and Send to “File” and click “Send to”.
- Open URL, e.g. http://localhost/PSE/index.php

- Input user name and click “Register”.

- Input Title, Keywords and Comment, select retrieved file and click “upload”. PSE displays “Registration completed!” after accomplishment. If the data is correctly registered, a directory of user name is created in PSE directory. If the directory is not created, please check the permission of PSE directory. In the user directory, data.txt, ori(ID).txt, mod(ID).txt, single(ID).txt, hitword(ID).txt will be included. If ori(ID).txt is not exist or the size of ori(ID).txt is zero, check the configuration of http server and PHP.

**Text Analysis**

- Open URL, e.g. http://localhost/PSE/index.php
-

- Input user name and click “Display”.
-

- Select dataset. Click “Gene” if you search sentence mainly with gene names, or click “Word” if mainly with common words. If you prefer to free text search, input text delimitating with space and click “Submit”. Free text search is “AND” search for all words. In this tutorial, click “Gene”.
-

- Select one gene symbol and common word. Parenthesis of each gene symbol and common word shows the count of sentences in the dataset. Select “Akt1” and click “Narrow_Down” here but if you directly go to the page showing sentence, click “Show_Sentence”
-

- Gene, “Akt1”, is fixed. The pull-down menu lists the cooccurrence words and the count of sentences. Select “SIGNALING” and click “Show_Sentences”.
-

- The result contains two parts, gene information part and sentence one. This is the gene information part. EntrezGene, RefSeq and OMIM information is linked from each ID. The homonyms are displayed by clicking symbol or alias. For example, click “PKB”.
-

- PKB represents three different genes. You can see the correct information in case of incorrect allocation if the correct gene is in the list.
-

- This is the latter part of the result. Selected gene name and keyword is highlighted. The number of each sentences is PubMed ID and link to whole abstract.
